# Incidence of obstetrical thrombotic thrombocytopenic purpura in a retrospective study within thrombocytopenic pregnant women. A difficult diagnosis and a treatable disease

**DOI:** 10.1186/s12884-015-0557-5

**Published:** 2015-06-17

**Authors:** Yahsou Delmas, Sébastien Helou, Pierre Chabanier, Anne Ryman, Fanny Pelluard, Dominique Carles, Pierre Boisseau, Agnès Veyradier, Jacques Horovitz, Paul Coppo, Christian Combe

**Affiliations:** Service de Néphrologie Transplantation Dialyse, Centre Hospitalier Universitaire de Bordeaux, Bordeaux, France; Centre de Compétence des Microangiopathies Thrombotiques, Centre Hospitalier Universitaire de Bordeaux, Bordeaux, France; Pôle Gynécologie-Obstétrique-et Médecine Foetale, Centre Hospitalier Universitaire de Bordeaux, Bordeaux, France; Service d’Hémostase Spécialisée, Centre Hospitalier Universitaire de Bordeaux, Bordeaux, France; Service d’Anatomie Pathologique, Centre Hospitalier Universitaire de Bordeaux, Bordeaux, France; Université Bordeaux Segalen, Bordeaux, France; Service de Génétique Médicale, Centre Hospitalier Universitaire de Nantes Hôtel Dieu, Nantes, France; Service d’hématologie, Centre Hospitalier Universitaire de Lariboisière, Assistance Publique Hôpitaux de Paris, Université Paris 7 Denis Diderot, Paris, France; Centre de Référence des Microangiopathies Thrombotiques, Paris, France; Service d’Hématologie Hôpital Saint Antoine, Assistance Publique Hôpitaux de Paris, Paris, France; Université Pierre et Marie Curie (UPMC), Univ Paris 6, Paris, France

**Keywords:** Pregnancy, Thrombocytopenia, HELLP syndrome, ADAMTS-13 deficiency, Thrombotic Thrombocytopenic Purpura, Upshaw-Schulman syndrome

## Abstract

**Background:**

Thrombotic thrombocytopenic Purpura (TTP) defined as ADAMTS-13 (A Disintegrin And Metalloprotease with ThromboSpondin type 1 domain 13) activity <10 % is a rare aetiology of thrombocytopenia during pregnancy, although the precise incidence is unknown. During pregnancy, the diagnosis of TTP is crucial as it has high feto-maternal morbidity-mortality and requires urgent plasma exchange. The purpose of this study was to assess the incidence of TTP retrospectively and to describe case presentations and follow-up.

**Methods:**

A monocentric retrospective study (2008–2009) was conducted among pregnant women followed in a tertiary care obstetrical unit who experienced at least one episode of severe thrombocytopenia (platelets ≤75 G/L) during 2008 and 2009. In cases of uncertain aetiology of thrombocytopenia, ADAMTS-13 activity was assessed by the full length technique.

**Results:**

Among 8,908 deliveries over the 2 year period, 79 women had a platelet count nadir ≤75 G/L. Eighteen had a known aetiology of thrombocytopenia and 11 were lost to follow-up. Among 50 remaining patients, ADAMTS-13 activity was undetectable (<5 %) in 4, consistent with the diagnosis of TTP. Platelet count spontaneously normalized in 3 patients after delivery. None presented focal cerebral involvement. Three of the four, who were primipara patients, had a sustained severe deficiency in the absence of anti-ADAMTS-13 antibodies, and ADAMTS-13 gene sequencing indicated a constitutive deficiency. The fourth, a multipara patient, had an acquired, auto-immune TTP. Placental pathology in the three primipara patients showed severe and non-specific ischemic lesions. Two patients lost their babies shortly after birth. In subsequent pregnancies in these two patients, prophylactic plasma infusion initiated early with increasing volume throughout pregnancy prevented TTP relapse, improved placental pathology, and led to normal delivery.

**Conclusions:**

The prevalence of TTP among thrombocytopenic pregnant women is high, up to 5 % in a tertiary unit. Platelet count normalization after delivery does not eliminate TTP. Clinicians should be aware of TTP during pregnancy, and, even if assessed retrospectively, ADAMTS-13 assessment is of particular importance for identifying patients with congenital TTP. In these patients, preventive plasma infusion and/or exchange can dramatically improve foetal prognosis, resulting in successful childbirth.

## Background

Thrombotic thrombocytopenic purpura (TTP) is a rare, life-threatening, thrombotic microangiopathy (TMA) characterized by severe deficiency in A Disintegrin And Metalloprotease with ThromboSpondin type 1 domain 13 (ADAMTS-13). This von Willebrand factor (vWF) cleaving protease deficiency is due either to autoantibodies directed against the protein in the acquired form of the disease, or to biallelic mutations of the encoding gene in the inherited form (also termed Upshaw-Schulman syndrome) [1]. In TTP, vWF and platelet-rich thrombi in small arterial vessels lead to mechanical haemolytic anaemia and thrombocytopenia with ischaemic injury of organs, preferentially in the brain and heart, which determines severity of the disease [1, 2]. Pregnancy is a known trigger of TTP; one precipitant might be that ADAMTS-13 activity physiologically decreases during pregnancy, while its substrate, vWF, increases [3–5]. Within pregnancy-related diseases, TTP can mimic other TMA such as Haemolysis Elevated Liver enzymes Low Platelet (HELLP) syndrome [6, 7]. It is critical to distinguish TTP from HELLP syndrome as TTP does not necessarily require pregnancy discontinuation [8–10] but does require urgent treatment based on plasma exchange [11]. The annual incidence of ADAMTS-13 deficiency TMA is less than 2 per million, and is more common in females [12]. Overall, the estimated incidence of TTP associated with pregnancy is less than 1 per 100,000 pregnancies [6], and before the era of ADAMTS-13 assays, it was found at a rate of 1 per 25,000 births [13]. The obstetrical incidence of TTP, as defined by a TMA with a severe ADAMTS-13 deficiency (activity <10 %) [14], has not been addressed in women during pregnancy or post-partum because the assay for ADAMST-13 activity has only recently become available. However, the identification of TTP during pregnancy is crucial, since it is associated with poor prognosis of both mother and foetus [5, 15]. To date, the assessment of ADAMTS-13 activity in pregnant women with thrombocytopenia has not been performed systematically, and the prevalence of TTP in pregnant women with thrombocytopenia remains uncertain. We conducted a two-year retrospective study at the Bordeaux University Hospital tertiary obstetrical unit. We assessed the incidence of TTP based on ADAMTS-13 activity in all consecutive pregnant or post-partum women presenting with thrombocytopenia less than 75 G/L. TTP patients were identified and followed through their subsequent pregnancies.

## Methods

Over a 2-year period (2008–2009), all consecutive patients hospitalized in the obstetrical unit who presented with at least one platelet count ≤75 G/L in the setting of pregnancy were considered. This threshold was used as a means to avoid over- or under-estimating TTP; ADAMTS-13 deficiency is associated with severe thrombocytopenia [16] and gestational thrombocytopenia is usually encountered above 70 G/L [7].

ADAMTS-13 activity was investigated in patients without obvious cause for low platelet count. If ADAMTS-13 activity was not previously measured or a historical blood sample was not available for testing, a new blood sample was obtained during normal post-partum follow-up. ADAMTS-13 assessment was performed as part of routine screening.

ADAMTS-13 functional activity was assessed by *full von Willebrand Factor length* technique as reported [17]. Activity <5 % was defined as a severe deficiency (activity >50 % is considered normal). ADAMTS-13 inhibitor was determined using a neutralizing inhibitor approach as previously described [17] and anti-ADAMTS-13 IgG was determined by ELISA (TECHNOZYME ADAMTS-13 INH, Technoclone, Austria). Our laboratory cut-off for a positive value was >35 IU/mL.

When ADAMTS-13 deficiency was found in repeated measures in the absence of inhibitor, genetic analysis was conducted for the 29 exons of the ADAMTS-13 gene and the exon-intron junctions as previously described [18]. These patients were followed prospectively and, during subsequent pregnancies, received virally inactivated fresh frozen plasma: 20–25 ml/kg fortnightly for the first trimester, weekly during second trimester, and 25–30 ml/kg weekly or plasma exchange for plasma volume over 1800 ml during the third trimester. Plasma amount was adapted to maintain normal platelet counts, normal levels of plasma LDH and haptoglobin. Residual activity of ADAMTS-13 was measured before each plasma infusion or exchange. Patients did not receive aspirin. Newborn weight percentile was assessed by the AUDIPOG tool [19].

Placentas were fixed in formalin, paraffin embedded, and stained by Haematein-Eosin-Safran.

This retrospective study was approved by the Comité de Protection des Personnes Sud-Ouest et Outre Mer III (ethics committee). All patients with ADAMTS-13 deficiency provided written informed consent for the publication of their individual clinical details.

## Results and discussion

**Fig. 1:**
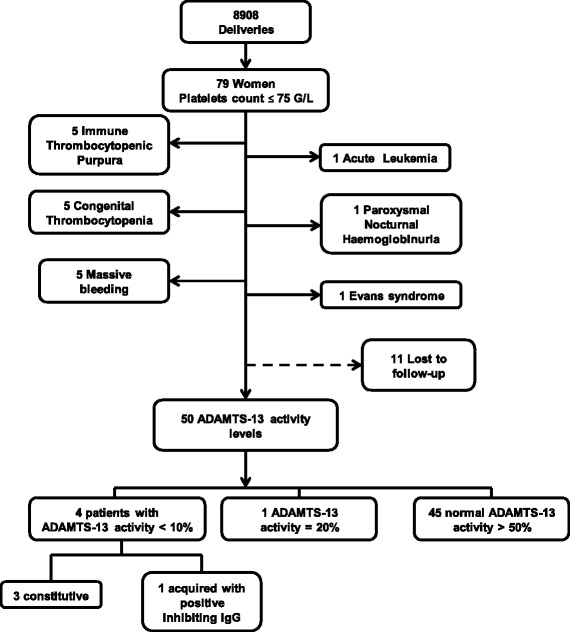
Characteristics of the patient cohort studied. In this retrospective analysis, all consecutive pregnant or post-partum patients seen in the obstetrical tertiary unit of Bordeaux university hospital who presented a platelet count ≤75 G/L were evaluated

Characteristics of the patient cohort studied are outlined in Fig. 1. There were 4,292 deliveries in 2008 and 4,616 in 2009 in the tertiary care obstetrical unit of Hôpital Pellegrin. Seventy-nine patients presented thrombocytopenia ≤75 G/L (43 and 36 in 2008 and 2009, respectively). Overall, 18 patients had a documented cause of low platelets, 5 with immune thrombocytopenic purpura, 5 with congenital thrombocytopenia (2 with MYH9 syndrome, 1 with von Willebrand disease 2B), 5 with massive delivery bleeding, 1 with paroxysmal nocturnal haemoglobinuria, 1 with acute leukaemia, and 1 with Evans syndrome with systemic lupus erythematosus and positive direct antiglobulin test. Eleven patients were lost to follow-up. ADAMTS-13 level was measured in 50 patients. One patient had an ADAMTS-13 activity of 20 % on retrospective analysis in the setting of intra-uterine foetal death due to retro-placental haematoma at 33 weeks of gestation (WG), with disseminated intravascular coagulation, a known condition of low ADAMTS-13 activity. She recovered spontaneously, and was lost to follow-up. Forty-five patients had normal ADAMTS-13 activity with a mean of 109 %.

Four patients without significant medical history had undetectable ADAMTS-13 activity in the peripartum period (Fig. 1). ADAMTS-13 activity assessment was mostly performed retrospectively from serum samples initially used for systematic viral serologies during hospitalization in the obstetrical unit. For some patients, ADAMTS-13 activity had been measured at the nephrologist post-partum consultation. Obstetric unit practitioners were unaware of this rare disease at the time patients were managed, and ADAMTS-13 assessment was not performed during pregnancy. Prior to the current systematic two year study, only one deficient patient was diagnosed at the post-partum consultation. Three primipara patients (Table 1, patients A, B, C) with an initial diagnosis of HELLP syndrome in the setting of low platelet count, who were identified the day of admission, were retrospectively suspected to have a constitutive severe ADAMTS-13 deficiency. This was confirmed on repeated ADAMTS-13 assays showing <10 % activity over multiple years in the absence of inhibitor. All three patients gave birth prematurely by caesarean section in emergency within 24 hours of hospitalization, with major placental lesions of ischaemia for all. The fourth patient (patient D) had a caesarean delivery for placenta praevia of her 7th pregnancy (gravida 7 para 3, with previous uneventful pregnancies except gravid hypertension during her first pregnancy); she had an episode of TTP on the 5th day post-partum, with presence of a plasma inhibitor and anti-ADAMTS-13 IgG antibodies at 70 IU/ml. She had positive anti-nuclear antibodies at 1/500 without anti-dsDNA. Of importance, she had received betamethasone at 29 WG and 31 WG for foetal pulmonary maturation because of bleeding. Patients’ clinical and biological characteristics are summarized in Table 1. All patients had an undetectable serum haptoglobin level at the time of thrombocytopenia. Schistocytes on blood smear were almost never assessed in the study patients. Lactate dehydrogenase (LDH) was not documented for patient C. LDH was slightly elevated in patients A and B, 1.3- and 2.1-fold over upper normal limits, and up to 4.0-fold in patient D, although not assessed daily. LDH/ASAT ratio, which suggests TTP rather than HELLP syndrome if over 22 [20], was 10, 2, and 56 for patient A, B, and D, respectively. Mean platelet count nadir for the 45 patients with ADAMTS-13 activity over 20 % was higher with mean (range) platelet count nadir of 53 G/L (16-75). For these patients, only one had a platelet count under 30 G/L (16 G/L at nadir, ADAMTS-13 activity of 54 %), but she had an uneventful pregnancy with vaginal delivery at 37 WG and no abnormal LDH, haptoglobin, or transaminase value. Patients A, B, C recovered spontaneously from their TMA without plasma infusion and exhibited normal blood TMA marker values at post-partum consultation. Patient D had asymptomatic thrombocytopenia of 46 G/L 23 days post-delivery but was lost to follow-up.

**Table 1:** Clinical characteristics of four pregnancy-related TTP patients

Patient	Age y.o.	Parity/Term of delivery	Child Sex/weight*/ outcome *kg-percentile	Smoking	Platelet nadir *G/L (Day from delivery)*	Clinical data Uricemia (μmol/L)	ADAMTS-13 activity (%) of patient’s Father/Mother (N: 50-150 %)
A	24	P0/28WG	Male	Yes	25	Headache	**22**/52
			1.3-38p		*(D + 2)*	Hypertension	
			Deceased			Proteinuria	
						Uricemia 441	
B	29	P0/33WG	Male	No	25	Headache	59/**34**
			2.2-30p		*(D0)*	No hypertension	
			Deceased			No proteinuria	
						Uricemia 250	
C	22	P0/31WG	Female	Yes	17	Headache	**15**/**43**
			1.17-0.1p		*(D + 1)*	Paresthesia	
			Healthy			Hypertension	
						Proteinuria	
						Uricemia 361	
D	35	P3/35WG	Female	Yes	24	Hypertension	Not Done
			2.9-50p		*(D + 5)*	Proteinuria	
			Healthy			Uricemia 194	

Patients A, B, C were primipara, whereas, patient D was expecting her fourth child. Patients A and B lost their babies in the neonatal period. For patient A, due to prematurity (enterocolitis at 6 weeks of age) and for patient B, probably because of a coarctation of the aorta and ventricular septal defect (post-partum day 18). All patients except patient B presented features of preeclampsia. Patient D, who presented post-partum auto-immune TTP, had two ADAMTS-13 activity assessments at 22 and 25 months after delivery which showed 11 % and 40 %, respectively, without inhibitor, but was lost to follow-up

(y.o.: years old; WG: weeks of gestation; G/L: Giga per liter; *D*: day of delivery)

**Fig. 2:**
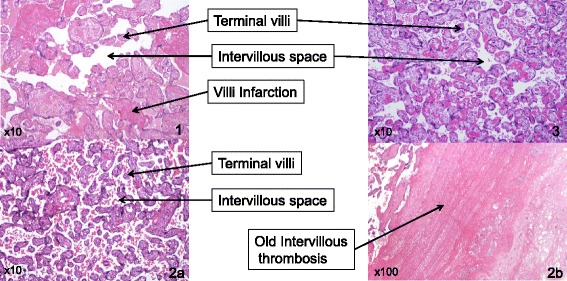
Patient B placental pathologies over 3 pregnancies with increasing plasma exposure. Placental pathologies of patient B over her three pregnancies are shown. 1. No plasma infusion in first pregnancy; delivery at 33 WG due to TTP with a significant advance in maturation and villi infarction over fibrin deposit. 2. Prophylactic plasma infusions from 13 WG in second pregnancy; significant advance in maturation and intervillous old thrombosis on placental pathology at 35 WG. 3. Plasma infusions from the beginning of her third pregnancy allowed birth at 38 WG and a placental histology consistent with the term. Second and third pregnancies were uneventful

In subsequent pregnancies patients A and B received prophylactic plasma infusion, and gave birth to healthy children. Patient B had 3 pregnancies overall: plasma infusion was initiated at 13 WG of her second pregnancy (upon retrospective diagnosis of TTP in her first pregnancy) and from 4 WG of her third pregnancy. In her second pregnancy she gave birth at 35 WG vaginally to a boy (birth weight 32^th^ percentile), and in her third pregnancy gave birth at 38 WG vaginally to a girl (birthweight 38^th^ percentile). ADAMTS-13 activity for patient B’s second and third babies were 33 % and 38 % after 6 months. The histopathological analysis of placentas showed an impressive improvement with plasma exposure during these three successive pregnancies (Fig. 2).

For patient A, similar observations were made; she received plasma infusion from the start of her second pregnancy and gave birth by planned caesarean section at 34 WG + 3 days to a healthy boy (ADAMTS-13 activity of 62 % at 8 months). Patient A received 3 plasma exchanges during the month preceding delivery because she needed large amounts of plasma (≥2 L). Residual ADAMTS-13 values ranged from 5 % before plasma therapy to 15 % throughout pregnancy with prophylactic treatment.

**Fig. 3:**
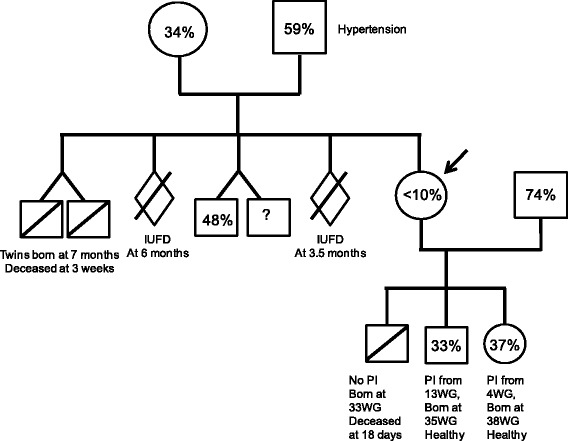
Patient B pedigree Arrow indicate the proband, patient B. Cercles and squares indicate females and males respectively. The percentages indicate known ADAMTS-13 basal activity. Activity for patient B children was assessed at respectively 6.5 for the youngest boy and 8 months old for the girl. (IUFD: intrauterine foetal death. PI: plasma infusion, WG: week gestation)

None of patients A, B, or C exhibited thrombophilia or autoimmunity. Patient A has a brother with ADAMTS-13 activity of 48 %. Patient C has 3 healthy younger sisters, two are adults with ADAMTS-13 activities of 29 % and <5 %. Patient B sibling history is detailed in a family tree (Fig. 3).

Genetic analysis of ADAMTS-13 for patients A, B, C are shown in Table 2. Patient A presented a heterozygous p.Arg1060Trp isolated mutation (this is patient n°24 in Moatti-Cohen *et al*. [18]). This isolated heterozygous status for the p.Arg1060Trp (c.3178C > T) mutation has been reported previously in late onset TTP triggered by pregnancy [21]. Potential deleterious effects associated with the p.Pro618Ala polymorphism in combination with other mutations in patients A and B have been described (see Table 2) [22]. No mutation was detected in any of the 29 exons of ADAMTS-13 in patient B, but there were four apparently homozygous polymorphisms that suggested a large deletion in one ADAMTS-13 allele. In concert with previously described polymorphisms occurring in patient B, this deletion may explain the low albeit detectable ADAMTS-13 activity during her non-pregnant status. In patient C, ADAMTS-13 was heterozygous for two mutations, one exon 6 sequence mutation affecting the protein structure and modifying the biochemical property of the metalloprotease domain and a second mutation located in intron 8 within the essential splice sequence of exon 9. The genetic analysis, clinical presentation, and familial history are indicative of a constitutive ADAMTS-13 deficiency in patients A, B, C.

**Table 2:** Genetic analysis of three patients with likely constitutive ADAMTS-13 deficiency

Patient	ADAMTS-13 gene mutation			ADAMTS-13 gene polymorphism Protein (status)	ADAMTS-13 activity N >50 %
	Exon/Intron number	Mutation (Status)	Protein	Protein	Non acute phase (basal)
A	Exon 24	c.3178C > T	p.Arg1060Trp	pArg7Trp (Htz)	5 %
		(Htz)	heterozygous	pGln448Glu (Htz)	
				*pPro618Ala* (Htz)	
				pAla732Val (Htz)	
				pAla1033Thr (Htz)	
B	-	-	-	pArg7Trp (Hm)	5-9 %
				pGln448Glu (Hm)	
				*pPro618Ala* (Hm)	
				pAla732Val (Hm)	
C	Exon 6	c.559G > C (Htz)	pAsp187His	-	<5 %
	Intron 8	c.988-2A > C (Htz)	not described		

Deleterious polymorphism are in italic [22]

Abbreviations: homozygous, Hm; heterozygous, Htz.

Obstetrical TTP is probably under-estimated, with an estimated incidence of <1/25,000 pregnancies [12,13]. In this study, the incidence for TTP in a tertiary obstetrical unit was nearly 1/2,000 pregnancies, and 1/20 among women with a platelet count ≤75 G/L. Despite the recruitment bias of a tertiary obstetrical unit, given our area of recruitment of 34,000 deliveries per year, we estimate the prevalence of TTP during pregnancy to be 1/17000 deliveries. This may also be an underestimate as there were 11 of 79 thrombocytopenic patients lost to follow-up. We confirmed the high rate of constitutive ADAMTS-13 deficiency in patients with obstetrical TTP, as observed by Moatti *et al.* in a previous report [18]. We also confirmed that pregnancy, by increasing the plasma levels of vWF, may trigger episodes of TTP in otherwise asymptomatic patients who carry specific ADAMTS-13 mutations, particularly the p.Arg1060Trp mutation [18, 21].

In this regard, three of our patients with constitutive, severe ADAMTS-13 deficiency recovered from TTP following delivery, without plasma infusion. Similar patients with spontaneous resolution of pregnancy-triggered TTP have been described by Fujimura *et al.* in Upshaw-Schulman families [15], and others since then have been reviewed in Veyradier *et al.* [5]. However, fatal TTP in the setting of pregnancy is commonly reported, and prompt plasma exchange is mandatory [23]. For the clinician, these observations suggest that thrombocytopenia during pregnancy requires (as in all thrombocytopenia) an assessment of haemolysis (LDH, haptoglobin) and a blood film examination. If symptoms consistent with the diagnosis of HELLP syndrome occur early during pregnancy and/or with severe thrombocytopenia, especially if there are features of placental ischaemia, ADAMTS-13 activity should be assessed, ideally in the acute phase of TMA. We found no obvious specific signs to distinguish HELLP from TTP; and, in the cases reported here, the ratio LDH/ASAT was not helpful [20], underscoring the possibility of misleading presentation [24]. Additionally, none of the TTP women had a history of jaundice, thrombocytopenia, or anaemia, and women with a severe constitutive deficiency were asymptomatic when not pregnant.

Identification of ADAMTS-13 deficiency is helpful to allow specific care [8, 9, 25–28] of this potentially fatal disease [10, 15, 23, 29–31]. In constitutively deficient women, as in previous reports, we invariably observed poor outcomes of pregnancy in the absence of plasma infusion prophylaxis. It is clear that the earlier plasma infusion is started the better the prognosis. The placental pathology in patient B supports this conclusion. Further studies to evaluate ADAMTS-13 deficiency in pregnancy and to identify biomarkers that would distinguish TTP from HELLP syndrome would be valuable. Until then, even when TTP presentation is mild, clinicians should ask for ADAMTS-13 activity analysis during the acute phase or at the latest, at the post-partum consultation or preconception consultation for subsequent pregnancies. If TTP is suspected, plasma exchange should be promptly initiated without waiting for ADAMTS-13 activity results.

## Conclusion

Clinicians should be aware of TTP in cases of early onset or severe thrombocytopenia during pregnancy, even if platelets normalize after delivery or if there is only mild haemolysis. In a tertiary obstetrical unit, The TTP incidence was more frequent than expected, approaching 1/2000 deliveries, and 1 per 20 thrombocytopenic patients ≤75 G/L. With respect to constitutive TTP, this study supports ADAMTS-13 activity assessment in the setting of HELLP syndrome, even done retrospectively, as this can clarify diagnosis, allowing prevention of maternal morbidity/mortality and leading to successful childbirth.

## Abbreviations

TTP: Thrombotic thrombocytopenic Purpura
ADAMTS-13: A Disintegrin And Metalloprotease with ThromboSpondin type 1 domain 13 TMA, Thrombotic microangiopathy
vWF: von Willebrand factor
HELLP: Haemolysis Elevated Liver enzymes Low Platelet
WG: Weeks of gestation
LDH: Lactate dehydrogenase
